# COVID-19 Vaccine Cardiac Complications: A Case Series on Implications of Marijuana in Adolescents With Myopericarditis

**DOI:** 10.7759/cureus.24665

**Published:** 2022-05-02

**Authors:** Sahil Zaveri, Helen Kest, Bhavin Shah, William DeBruin, Mario Colletti

**Affiliations:** 1 Pediatrics, St. Joseph's Children's Hospital, Paterson, USA; 2 Pediatric Infectious Diseases, St. Joseph's Children's Hospital, Paterson, USA; 3 Pediatric Intensive Care Unit, St. Joseph's Children's Hospital, Paterson, USA

**Keywords:** chest pain, vaccination, marijuana, covid-19, myopericarditis

## Abstract

We report on two critically ill pediatric patients, aged 16 and 18 years, presenting with acute myopericarditis at a tertiary-care center in New Jersey, United States. Both patients had their severe acute respiratory syndrome coronavirus 2 (SARS-CoV-2) vaccinations, tested negative for SARS-CoV-2, and shared only significant history of asthma. Clinical presentations were similar to acute onset chest pain that worsened with deep inspiration. One patient reported a history of vaping and escalating marijuana use several hours preceding presentation. Both patients had elevated troponin on admission and had ST-segment elevation on electrocardiogram (EKG), thus prompting admission to the pediatric intensive care unit (PICU) for cardiac monitoring. Myopericarditis has multiple etiologies and is a newly described rare complication of the SARS-CoV-2 vaccine. It can also occur as a complication of vaping and frequent marijuana drug use. Our paper highlights the importance of a detailed social and drug history in adolescents presenting with chest pain. The clinical characterization is necessary to promote better case definitions and the design of targeted interventions for this vulnerable group.

## Introduction

In response to the global pandemic of coronavirus, COVID-19 vaccinations were developed and produced as a primary preventive measure to help mitigate the spread and severity of those affected. Vaccinations have been monumental in preventing the transmission of infections in both the school and community [1]. Myopericarditis had become a documented sequelae of the SARS-CoV-2 vaccine [2]. Etiologies of myopericarditis can be divided into infectious, mainly viral, and non-infectious, which include, but are not limited to, drugs, medications (hydralazine), alcohol, heavy metal poisoning, post-radiation, systemic inflammatory states, cancers, metabolic diseases, and vaccine associations [3]. Adolescence is often a period where there is a stark increase in drug experimentation, especially marijuana. It also represents a period where targeted intervention in the form of mitigation strategies can promote the prevention of several complications that may carry into late adulthood. We describe two pediatric patients with myopericarditis post-vaccination presenting to a tertiary-care center in New Jersey. Both patients were stable while in the PICU, had cardiology consultations, trending troponins, and echocardiograms (ECHO) which showed no abnormalities. Both were diagnosed with myopericarditis. They recovered well and were discharged shortly after admission.

## Case presentation

Case 1

**Figure 1:**
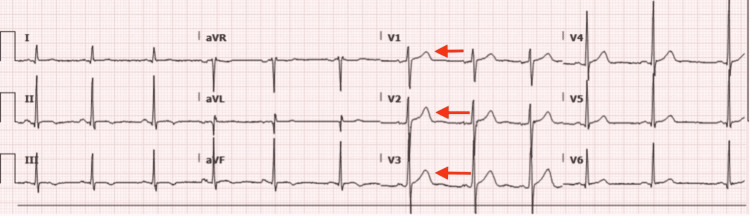
EKG on admission. Electrocardiogram (EKG) revealed diffuse ST-segment elevation with an absence of ST-segment depressions.

An 18-year-old previously healthy male with a past medical history of asthma presented to the emergency room for a sudden onset of chest pain. The chest pain started 30 minutes before arrival while he was vaping marijuana. He reported using increasing amounts of cannabis and vaping in the preceding few days. The pain was moderate to severe, pleuritic on the anterior chest wall, worsened with deep breaths, and was non-radiating and non-exertional in nature. He took an aspirin without any relief. He had no known sick contacts or recent foreign travels. In the emergency department (ED), vitals were temperature of 36.6°C, heart rate of 68 beats per minute (pm), respiratory rate of 20 breaths pm, blood pressure 120/85 mmHg, and oxygen saturation of 94%. Laboratory workup showed elevated troponin 1,070 pg/mL at 12:15 pm, and two hours later, it increased to 3,362 pg/mL, and ST-segment elevation was appreciated on the EKG (Figure 1). Urine toxicology reports revealed marijuana was positive, and SARS-CoV-2 polymerase chain reaction (PCR) was negative. He had received his Pfizer COVID-19 vaccination two months prior to the presentation. He was transferred and admitted to the PICU. While in the PICU, the patient was assessed by the cardiology team and diagnosed with myopericarditis. A repeat EKG was done two days later and was within normal limits. Echocardiogram was insignificant. While in PICU, troponin levels trended down. The patient was hemodynamically stable on the second day of admission, and the pain was rated 0/10. The patient responded to supportive measures, including treatment with Ibuprofen, and was discharged in stable condition. Follow-up with cardiology four weeks post-hospitalization showed that he was doing well with unremarkable EKG and repeat ECHO. 

Case 2

**Figure 2:**
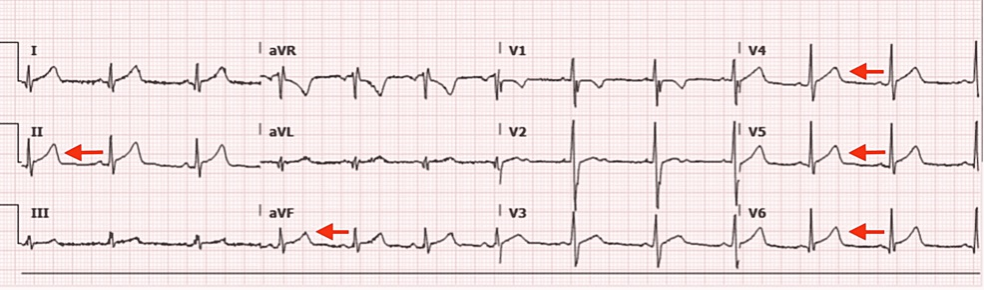
EKG on admission. Electrocardiogram (EKG) revealed ST-segment elevations in V3-V6 anteriorly, and T-wave inversions in the inferior leads.

A 16-year-old, previously healthy male with a past medical history of asthma presented to the emergency department (ED) with complaints of chest pain. He stated the pain started in the early morning hours and was mild, intermittent, and non-radiating in nature. It was aggravated by deep breaths and was alleviated with time and rest. The symptoms initially resolved but returned in the late morning, which prompted his mother to bring him to the emergency room. There was no active chest pain in the ED. Of note, the patient received a second dose of his Pfizer COVID-19 vaccine four days prior. There were no known sick contacts or recent foreign travels. The patient denied any extremity pain or swelling, any previous chest pain, or other associated symptoms at this time. In the ED, the vitals were: temperature 36.5°C, heart rate 79 beats pm, respiratory rate 20 breaths pm, blood pressure 125/62 mmHg, and oxygen saturation 99%. Pertinent lab findings included elevated troponin 7,367 pg/mL at presentation. EKG revealed ST-segment elevations in V3-V6 anteriorly, and T-wave inversions in the inferior leads (Figure 2). The patient was transferred and admitted to the PICU with a diagnosis of myopericarditis. EKG showed improvement with decreased ST elevation on day two of admission. The echocardiogram at this time was normal without any signs of pericardial effusion. The following morning, troponin levels had peaked at 12,085 pg/mL on day three. The fourth morning's labs demonstrated a marked increase in troponin again to 10,081 pg/mL, but the patient was asymptomatic. After the patient’s troponins decreased below 6,000 pg/mL, the patient was scheduled for a follow-up outpatient visit and was discharged on the fifth-day post-admission. Follow-up with cardiology four weeks post-hospitalization showed that he was doing well with unremarkable EKG and repeat ECHO. Clinical characteristics and laboratory evaluation are summarized in Table 1.

**Table 1:** Clinical characteristics and laboratory evaluation of patients. SARS-CoV-2: severe acute respiratory syndrome coronavirus 2, RT-PCR: reverse transcription polymerase chain reaction, ICU: intensive care unit

	Patient 1	Patient 2
Age	18	16
Sex	Male	Male
Race	Hispanic	Hispanic
Time to presentation (hours)	1	6
SARS-CoV-2 testing (RT-PCR)	Negative	Negative
Days since SARS-CoV-2 Vaccination (days)	60	4
Laboratory values		
Leucocytes (K/μL) (4.5–13.5)	5.9	12
Platelets (K/μL) (140–440)	202	273
Neutrophils (K/μL) (1.30–9)	3.83	8.28
Lymphocytes (K/μL) (1.90–7.5)	1.29	1.94
C-reactive protein (mg/L) (<9.9)	8.2	55.4
Erythrocyte sedimentation rate (mm/hour) (0–20)	4	50
Fibrinogen (mg/dL) (183–503)	307	396
D-dimers (mcg/mL) (≤0.5)	0.23	<0.22
Ferritin (ng/ml) (13–145)	40	87
Albumin (g/dL) (3.8–5.4)	4.7	4.5
Creatinine (mg/dL) (0.6 to 1.3)	0.89	0.81
Troponin (ng/mL) (0.00–0.030)	3,592	7,367
B-type natriuretic peptide (pg/mL) (1–100)	50	52
Creatine kinase (unit/L) (30-223)	359	N/A
Chest X-ray imaging	Unremarkable	Unremarkable
Electrocardiogram	Diffuse ST-segment elevation	ST-segment elevation in V3-V6 anteriorly and T-wave inversions in inferior leads
Transthoracic echocardiography	1. Left ventricular ejection fraction is normal 55%-60%. 2. Mild tricuspid regurgitation.	1. Left ventricular ejection fraction is normal 55%-60%. 2. Trivial pulmonary valve regurgitation and tricuspid valve regurgitation.
Duration of ICU stay (days)	2	2

## Discussion

Here we report on two cases of myopericarditis in a tertiary care center in New Jersey with two adolescent patients after receiving their second dose of the mRNA COVID-19 vaccine at four days and 60 days post-vaccine. Patients were otherwise healthy, developed severe chest pain, and underwent intensive care management. 

Myopericarditis cases have been reported to the vaccine adverse event reporting system (VAERS), a part of the Centers for Disease Control and Prevention (CDC), which tracks adverse events due to vaccinations. It remains a rare side effect in young adolescents and is more likely to occur after the second dose [4]. Many cases have occurred in young adolescent males within a week of receiving their second vaccination dose. Cases can continue to occur up to 90 days post-vaccination. These patients have recovered quickly with rest, medication, and supportive management [1-4].

Myopericarditis is a constellation of symptoms, including high troponins and chest pain with an inflammation of the myocardium and pericardium, usually as a complication of acute pericarditis. It is interchangeably used with perimyocarditis [2,3]. The primary identification is by laboratory biomarkers and imaging studies which are used in other forms of carditis with the utilization of troponins, creatine kinase, electrocardiogram, and echocardiogram [3].

Both patients presented with chest pain, the most common presenting complaint. Both patients had recently received their second dose of the COVID-19 vaccine, similar to the findings in a detailed cross-sectional study [5]. The patient from case 1 had a positive history of escalating vaping and marijuana use, representing an alternate etiology. The patient from case 2 had received his second dose just a few days prior to developing complaints. The prolonged hiatus between vaccine and onset in case 2 also supports this alternate etiology as many vaccine-related cases occur within the first week.

Both patients were admitted to the pediatric intensive care unit for continuous cardiopulmonary monitoring, including trending troponin, brain natriuretic peptide, erythrocyte sedimentation rate, and C-reactive protein every eight hours. They were supportively managed with acetaminophen, Ibuprofen, famotidine, lidocaine, and intravenous fluids [6]. Both patients recovered promptly and were at pre-morbid baseline on outpatient follow-up. Well-known etiologies of myopericarditis are marijuana inhalation, viral, autoimmune, neoplastic lesion, post-infarction, and post-vaccination [5,6].

Marijuana is one of the most frequently used illicit substances in adolescence [7]. Vaping in adolescence is increasingly used to deliver cannabis and other psychoactive substance, with about one in three high school seniors engaging in its use [8]. In addition to these complications, marijuana-induced cardiovascular events include myopericarditis, arrhythmias, cerebrovascular disease, peripheral artery disease, and metabolic changes such as a decrease in low-density lipoprotein, and fasting glucose levels [9-12]. Cardiac changes seen in our patient in case 1 presented acutely while vaping with chest pain, elevated troponins, ST-segment changes, and a positive drug screen for marijuana. Proposed mechanisms by which marijuana may cause carditis are platelet aggregation and adhesion, coronary vasospasm, and arteritis [11]. These are thought to occur due to the various cannabinoid receptors CB1/CB2, and their vast presence throughout the body [12]. Marijuana can alter physiology by the mechanism of cannabinoid receptors in the autonomic nervous system, as this allows cannabis to affect blood pressure and heart rate [11-13].

Though there are limited research studies on time to heart disease presentation with cannabis use, one study showed a decreased time of onset for chest pain after cannabis use [13]. Myopericarditis is a rare complication that occurs after mRNA COVID-19 vaccination (Pfizer-BioNTech or Moderna), especially in male adolescents and young adults. It most often occurs after the second dose in male adolescents. Outcomes and recovery are typically good. We recommend that all adolescents presenting with chest pain any time post-COVID-19 vaccine receive an in-depth evaluation for other differentials, which should include a detailed history of vaping marijuana and other drug use. If positive, a team of social services, caregivers, and the adolescent’s physician should recommend valuable resources and intervention that targets the underlying triggers [11-13]. 

## Conclusions

COVID-19 vaccine has undoubtedly reduced the worldwide disease burden. Together with other preventive measures, vaccines have helped curb the ever-evolving COVID-19 pandemic. Myopericarditis is a rare side effect in adolescents with typically good outcomes. Our study highlights the essence of good history taking, which helps broaden differentials and understanding of other synergistic factors with the goal of promoting targeted interventions in this vulnerable group. For an adolescent presenting with chest pain attributable to myopericarditis, a detailed history taking is crucial to developing a comprehensive differential diagnosis. It is imperative to inquire about a thorough history of drug abuse, especially marijuana use.
